# Antisolvent Crystallization of Telmisartan Using Stainless-Steel Micromixing Membrane Contactors

**DOI:** 10.1021/acs.cgd.3c00123

**Published:** 2023-04-24

**Authors:** Matthew John Bennett, Elina Beveniou, Alex Robin Kerr, Marijana M. Dragosavac

**Affiliations:** † Wilton Centre, Micropore Technologies Ltd, Redcar TS10 4RF, U.K.; ‡ Chemical Engineering Department, Loughborough University, Leics LE11 3TU, U.K.

## Abstract

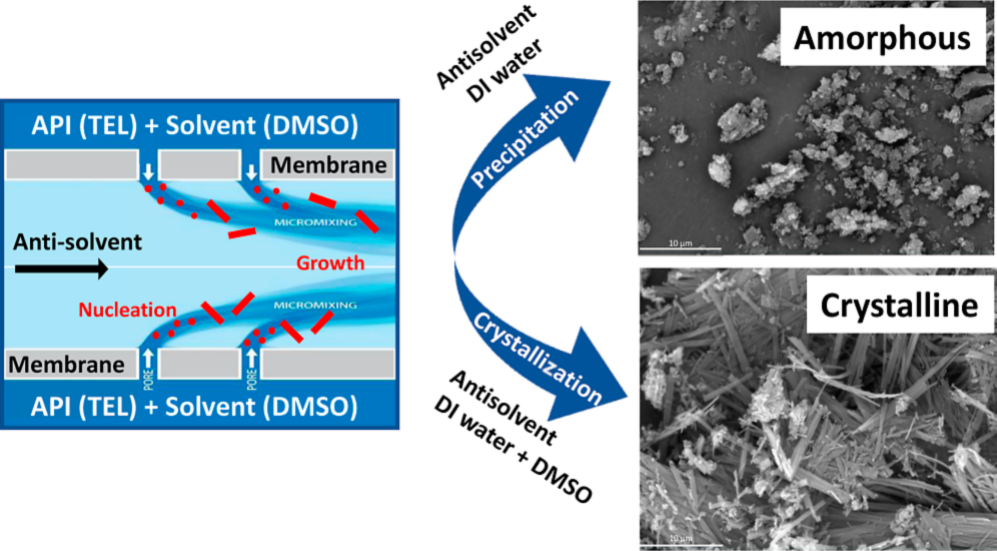

Controlled continuous crystallization of the active pharmaceutical ingredient (API) telmisartan (TEL) has been conducted from TEL/DMSO solutions by antisolvent crystallization in deionized water using membrane micromixing contactors. The purpose of this work was to test stainless-steel membranes with ordered 10 μm pores spaced at 200 μm in a stirred-cell (batch, LDC-1) and crossflow (continuous, AXF-1) system for TEL formation. By controlling the feed flow rate of the API and solvent, through the membrane pores as well as the antisolvent flow, it was possible to tightly control the micromixing and with that to control the crystal nucleation and growth. Batch crystallization without the membrane resulted in an inhomogeneous crystallization process, giving a mixture of crystalline and amorphous TEL materials. The rate of crystallization was controlled with a higher DMSO content (4:1 DMSO/DI water), resulting in slower crystallization of the TEL material. Both membrane setups, stirred batch and the crossflow, yielded the amorphous TEL particles when deionized water was used, while a crystalline material was produced when a mixture of DI water and DMSO was used.

## Abstract 2

Controlled continuous crystallization of the API telmisartan (TEL) has been conducted from TEL/DMSO solutions by antisolvent crystallization in deionized water using membrane micromixing contactors. The purpose of this work was to test stainless-steel membranes with ordered 10 μm pores spaced at 200 μm in a stirred cell (batch, LDC-1) and crossflow (continuous, AXF-1) system for TEL formation. By controlling the feed flow rate, of the API and solvent, through the membrane pores as well as the antisolvent flow, it was possible to tightly control the micromixing and with that control the crystal nucleation and growth. Batch crystallization without the membrane resulted in an inhomogeneous crystallization process giving a mixture of crystalline and amorphous TEL material. The rate of crystallization was controlled with a higher DMSO content (4:1 DMSO/DI water) resulting in slower crystallization of the TEL material. Both membrane setups, stirred batch and the crossflow, yielded the amorphous TEL particles when deionized water was used, while the crystalline material was produced when a mixture of DI water and DMSO was used.

## 1 Introduction

**Figure 1:**
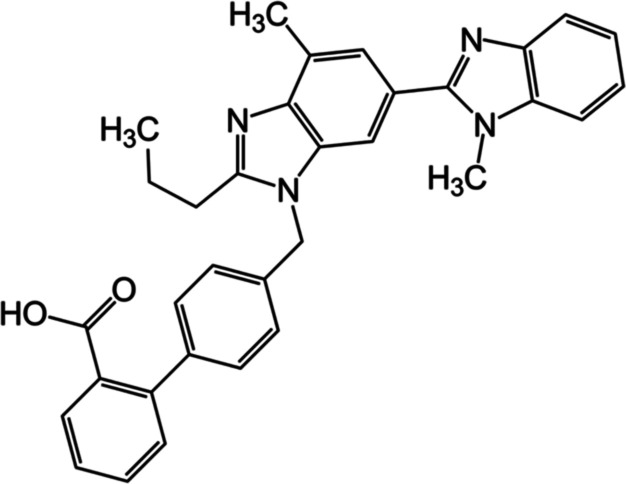
Molecular structure of TEL (C_33_H_30_N_4_O_2_), a BCS type II angiotensin II receptor antagonist.^1^

Telmisartan (TEL) (Figure 1)^1^ is an angiotensin II receptor antagonist, often used to treat hypertension and heart failure. According to the Biopharmaceutical Classification System (BCS), it is classified as a type II drug and therefore exhibits poor dissolution rates,^2^ water solubility (9.9 μg/mL),^3,4^ and bioavailability (40–58% dosage-dependent)^5^ similar to many active pharmaceutical ingredients (APIs). Such characteristics pose an issue when it comes to dosage formulations. As such, it has become increasingly common to reduce the crystal particle size and hence increase its surface area to improve solubility and bioavailability.^6,7^ Furthermore, the amorphous form of an API often exhibits better dissolution and bioavailability compared to its crystalline counterpart.^2,8^

Various methods have been used to decrease the particle size of API crystals, such as milling, spray drying, sonication, and high-pressure homogenization.^3,9^ However, such techniques are energy-intensive, not cost-effective, often result in poor batch-to-batch reproducibility^6^ as well as thermal degradation,^9^ and have broad crystal size distribution (CSD).^10^

Cooling crystallization is the most common method used in industry for the improvement of solubility and can be performed as a batch and continuous process.^11^ While advantageous in its simplicity, cooling crystallization can be an expensive and a time-consuming process with poor control, often resulting in the production of large crystals with wide and uncontrolled CSDs.^12^ Cooling crystallization also poses the risk of thermal degradation as the API is often kept at high temperatures for a prolonged period^3^ during temperature cycling. Antisolvent crystallization is a rapid alternative which involves the mixing of an API (solute) containing a primary solvent with a secondary miscible solvent known as the antisolvent. This results in a reduction of the solubility of the API (solute) in the primary solvent leading toward supersaturation. Properly controlling the supersaturation of the API can lead to high levels of nucleation as the metastable zone limit is exceeded, resulting in smaller crystals with tight and defined CSDs.^14,15^ Antisolvent crystallization can be readily scaled up^16^ and presents great potential for the industrial production of APIs.

Most of the pharmaceutical industry carries out crystallization of APIs via batch processes;^17^ however, this method comes with disadvantages such as poor batch-to-batch reproducibility and high production costs.^15,18,19^ The benefits of continuous production in the pharmaceutical industry range from a faster transition from API development to launch scale production, greater control and easier isolation of potential production faults,^20,21^ reduction of stock, and reduced dependence on external imports.^22^

It has also been estimated that even in the case of poorer yields through a continuous process, overall cost savings can still be achieved compared to a batch process.^23^ Some examples of continuous crystallizers include plug flow tube systems^23^ which can easily foul without seeding,^25^ continuous oscillatory baffled crystallizers,^26^ which can be complicated to use and take up a large amount of space, and mixed suspension crystallizers used for mixed product removal.^27^ These are the most used continuous crystallizers which still suffer from broad crystal residence time distribution and thus broad CSDs. These crystallizers often use cooling or antisolvent crystallization or a mix of both. Problems with back-mixing, slurry transport, and the inconvenience of handling equipment with moving parts are some of the main reasons that have prevented continuous processes from being the preferred choice.^28,29^

Besides the crystal size and CSD, the range of order (crystallinity) of the API also influences the solubility. Due to its high free energy and low-range order, the amorphous phase has high aqueous solubility; however, these phases have the potential to recrystallize toward the more stable crystalline form.^30^ The amorphous forms of APIs are advantageous within the pharmaceutical industry due to their higher solubility.^31−34^ This is particularly important for type II APIs including TEL which have low aqueous solubility.^4^ It has been possible through controlling the supersaturation conditions to selectively crystallize TEL in the amorphous phase for pharmaceutical needs.^35−37^

In this study, batch and continuous membrane systems, previously used to produce monodisperse emulsions, nanoparticles, liposomes, and piroxicam crystals,^6,38−40^ have been used in the crystallization of TEL. Stirred batch cell (LDC-1) systems, where a mixing shear is provided by a paddle stirrer suspended over a disc membrane, and a crossflow continuous device (AXF-1), where mixing shear is provided with the liquid flow through the center of a tubular membrane, have been investigated for TEL crystallization. Both units rely on passing the API-solvent-rich phase through a laser-drilled porous, stainless-steel membrane into an antisolvent phase. Laminar flow of the antisolvent phase across the membrane surface ensures even distribution of the API-solvent-rich phase pushed through the pores, resulting in precise and reproducible mixing.^32,41^ The high membrane throughput due to straight-through pores and robust construction as well as high porosity quickly supersaturates the antisolvent, leading to high levels of nucleation and the production of large amounts of micron-sized uniform crystals.^42^

This TEL crystallization study, without additional stabilizers, acts as a principal example of how membrane technology and micromixing can be applied for antisolvent crystallization across batch to continuous devices (LDC-1 and AXF-1) using different methods to provide mixing shear (stirring and fluid flow). Rapid mixing provided by this technology to control particle size, CSD, and crystallinity was also investigated. Conditions which direct the crystallization toward the amorphous phase, which is less thermodynamically stable but more soluble^43,44^ compared to the crystalline phase, have been determined.

## 2 Experimental Section

### 2.1 Chemicals

TEL, the API under study (99% purity), was purchased from Combi-Block (San Diego, USA). The solvent was dimethyl sulfoxide (DMSO) (99.7% pure) from Acros Organics, Thermo Fisher Scientific (Geel, Belgium), and the antisolvent was deionized (DI) water.

### 2.2 TEL Crystallization in a Batch Stirred-Cell (LDC-1) Membrane Micromixing Setup

**Figure 2:**
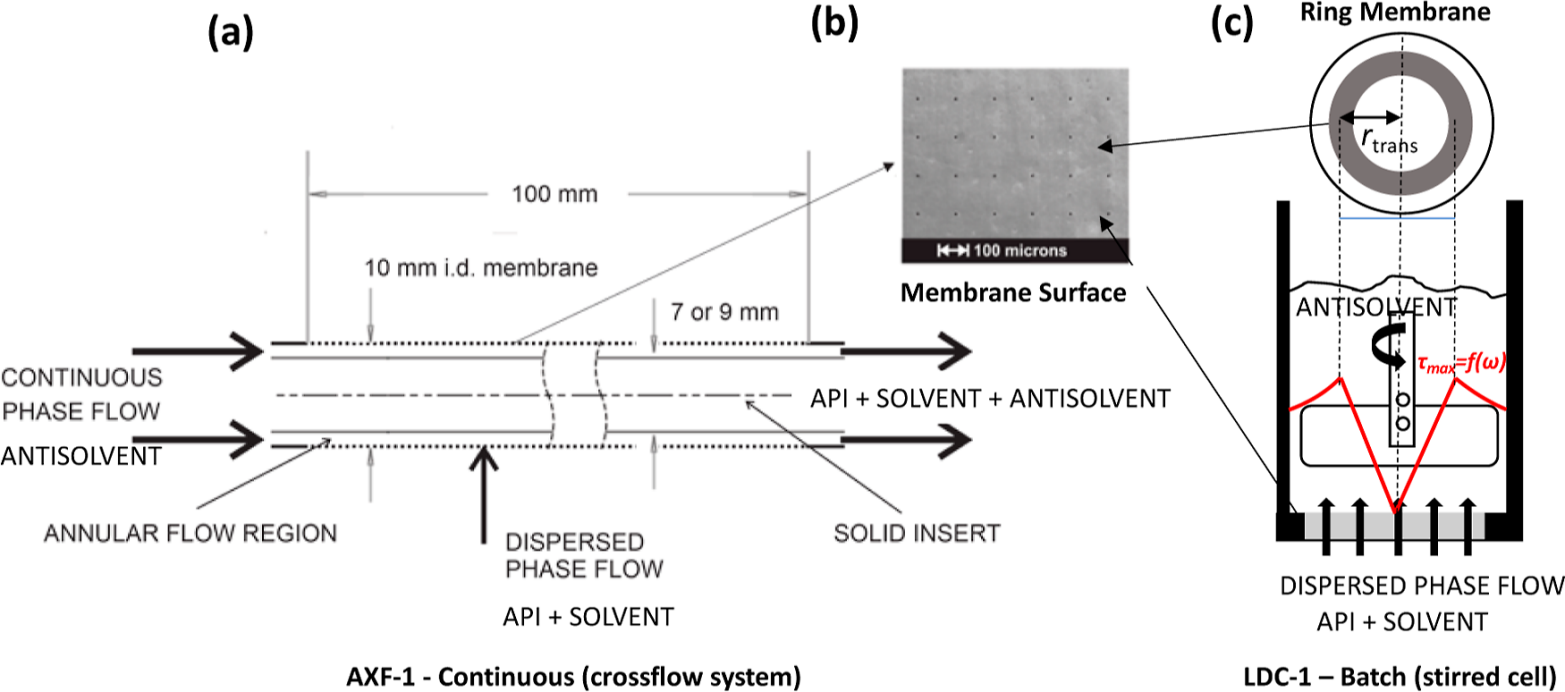
Schematic illustration of batch and continuous membrane systems. (a) AXF-1 crossflow annular flow system with a tubular membrane (left). (b) Stainless-steel membrane surface with drilled holes (middle). (c) Stirred-cell (LDC-1) setup with the paddle above the stainless-steel (SS) ring (right).

In the LDC-1 (manufactured by Micropore Technology, Redcar, UK) batch stirred cell,^37,45^ the API/solvent mixture was injected through a ring membrane into the antisolvent, and mixing between the API/solvent and antisolvent was promoted with the stirrer, positioned in the cell at a fixed distance (6 mm), above the stainless-steel (SS) membrane (Figure 2). The ring membrane had approximately 6900 cylindrical pores of 10 μm diameter fabricated by laser ablation arranged within the *Ar* = 2.76 cm^2^ ring area with a 200 μm spacing between the pores.

A ring membrane was chosen so that the shear that detaches the droplets from the membrane surface stays constant, allowing equal droplet/stream formations from the evenly positioned pores with a set of pore radius  values. The maximal shear  is determined by eq 1
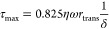
1

The LDC-1 was filled with 50 mL of the continuous phase (CP) antisolvent which in each case was DI water. Different stirrer speeds resulting in different shear stresses at the membrane surface were tested, 14.9, 24.6, and 35.7 Pa. Of these different shears, 24.6 Pa gave TEL particles of the tightest size distribution showing the most homogeneous CSD.

**Figure 3:**
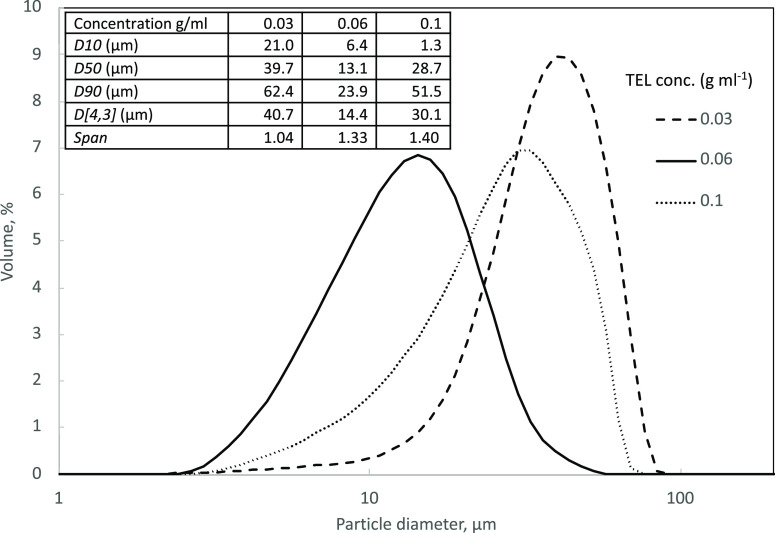
CSD curves of TEL particles from batch stirred-cell crystallization using different concentrations with a stirrer shear stress of 24.6 Pa and a flow rate of 10 mL min^–1^. The inserted table shows the corresponding diameters, mean, and span of TEL particles from batch stirrer-cell crystallizations.

A study of TEL/DMSO solutions with different TEL concentrations (0.03, 0.06, and 0.1 g mL^–1^) injected through the membrane was carried out. Of these values, 0.06 g/mL showed the smallest size crystals, while 0.03 g mL^–1^ gave the tightest distribution (Figure 3). Precipitation and volumetric productivity increased with higher TEL concentration in the dispersed phase and ranged between 3.1 and 10.6 mg mL^–1^.

The TEL/DMSO solution was preheated to 70 °C to achieve full API dissolution in DMSO and was kept at 70 °C to prevent crystallization during the injections. AL-300 World Precision Syringe Pumps (World Precision Instruments Ltd, UK) with a syringe heater were used to maintain the TEL/DMSO temperature, and an injection rate of 10 mL min^–1^ was used to also prevent crystallization during the injection. Each run added 6 mL of the dispersed phase (API + Solvent) to 50 mL of DI water (antisolvent) in the stirred cell. From the API solubilities in the solvent and antisolvent (assuming full mixing), TEL supersaturation in LDC-1 was estimated to be 0.11598 g L^–1^. During the experiments, the antisolvent was not heated and was kept at room temperature (20 °C) to achieve rapid supersaturation and precipitation. This was visually confirmed upon addition of the TEL/DMSO mixture into the water (antisolvent), resulting in the overall solution turning turbid white. It is worth noticing that the solubility of TEL increases with temperature; hence, after the full addition of the TEL/DMSO mixture, stirring was continued for 10 min, resulting in the suspension cooling to 20 °C before sample collection.

To represent the droplet/stream of the TEL/DMSO mixture being injected through the membrane, an estimate of the initial diameter of the droplet formed at the membrane surface *x* was calculated from the force balance of the retaining (capillary force) and detaching forces (drag force) acting on a single droplet at a single membrane pore, eq 2
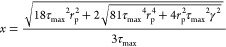
2Here, *r*_trans_ is the transitional radius that describes the vortex around the membrane where mixing changes from a forced vortex to a free vortex, η is the dynamic viscosity of the CP, ρ is the CP density, ω is the angular velocity, and δ = μ/(ωρ) is the boundary layer thickness.^45^

The maximum shear stress determined is then applied within eq 1 to provide a prediction of the droplet size. The latter is then compared with the experimental values obtained for different shear stress and CP viscosity conditions investigated.

### 2.3 TEL Crystallization in a Continuous Crossflow (AXF-1) Membrane Micromixing Setup

The annular crossflow membrane system used in this work was set up as illustrated in Figure 1 (left). The dispersed phase (DP) was preheated to 70 °C and stirred at 300 rpm continuously before and during its addition through the vertical inlet. The CP DI water would initially be run through the horizontal inlet of the continuous crossflow cell covering the membrane before the DP was added. The CP would flow through the system at room temperature and without the need for stirring. Control of the DP and CP flow rates was carried out using 2 ISMATEC MCP-Z gear pumps (Cole-Parmer GmbH, Germany) with Cole-Parmer P/N 07001–40 pump heads (Cole-Parmer GmbH, Germany). Different CP and DP flow rate combinations were investigated for their effects on the size and CSD. Stainless-steel membranes used had pore diameters of 5, 10, 20, and 40 μm, all with a square pitch of 200 μm [Figure 1(middle)].

The continuous crossflow equipment used is an annular flow single-pass crossflow (AXF) membrane emulsification system (manufactured by Micropore Technology, Redcar, UK). This setup arrangement consists of a tubular membrane with a 10 mm internal diameter and a 100 mm active membrane length. Insert rods can be used to vary the shear, while flow dividers and receivers split and evenly distribute the incoming (and leaving) CP flow within the annular flow channel that lies next to the inner surface of the membrane (Figure 1). The shear rate in the continuous (crossflow) system works the same as in the stirred batch cell (LDC-1) with the only difference being that it is the flow rate that controls the shear and not the stirring rate.

The total height of the annular flow channel can be varied to 1.5 or 0.25 mm, and during the crystallization experiments, it is maintained at 0.25 mm. The DP phase is introduced into the gap between the outer surface of the tubular membrane and the inner surface of the shroud. All metal components are made of stainless steel and are suitable for in-process sterilization.

In a single-pass annular flow system,^40,46^ the volumetric flow rate (*Q*) and pressure gradient axially (*−*d*P/*d*z*) within the annular region are described by eq 3.^32,38^
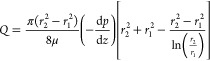
3where *r*_2_ is the radius of the outer annulus wall (i.e., the membrane), *r*_1_ is the radius of the inner annular wall (i.e., the insert radius), and μ is the coefficient of liquid viscosity. The pressure gradient is obtained from a rearranged version of eq 3 if the volume flow rate, liquid viscosity, and geometry of the tubular system are known. The wall shear stress at the surface of the membrane (outer annulus wall) is defined in eq 4
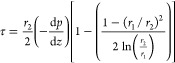
4Thus, the shear stress at the surface of the membrane can be determined from eq 2 using the pressure gradient from the rearranged form of eq 3.

During the annular crossflow, the DP and CP will interact to form an overall flow pattern. The flow pattern can be understood by calculating the Reynolds number, *R*_*e*_.^4,6^ The Reynolds number is the ratio of the inertial forces to the viscous forces within a fluid which is subjected to relative internal movement due to different fluid velocities. A stable and symmetrical flow is preferred over an unstable turbulent flow. The Reynolds number of the overall annular flow can be determined via eq 5 using *d*_h_ as the hydraulic diameter of the membrane, *u* as the crossflow velocity, and μ and ρ as the feed density and viscosity, respectively.
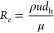
5

In the crossflow membrane system, supersaturation occurred immediately upon the interaction of the DP with the CP in the annular flow and turbidity was observed in the collection beaker. The output mixture was stirred using a magnetic stirrer bar at 300 rpm until approximately 100 mL (CP + DP) would be collected. The solution would then be stirred for a further 10 min before analysis of crystal size and morphology would be carried out using laser diffraction and optical imaging.

For further analysis, vacuum filtration of the resulting slurry was carried out over cellulose-based filter paper with either 2.2 or 8.0 μm pores, and the slurry was continuously washed with excess DI water to remove any residual DMSO. The resulting TEL was dried using a box furnace at 60 °C for up to 3 h before being collected for X-ray powder diffraction (XRPD), FTIR spectroscopy, and scanning electron microscopy (SEM) analysis.

All experiments for batch and continuous crystallization used triplicates, and the average size and CSD have been reported.

### 2.4 Characterization Techniques Used in the Work

#### 2.4.1 Crystal Size Distribution

The CSD was measured by laser diffraction using a Beckman Coulter LS230 (Wycombe, UK). A saturated aqueous solution of TEL (concentration >9.9 μg/L in water) was used as a dispersant. The relative volume, *V*_*i*_, of the particles in different size classes *i*, with mean diameter *d*_*i*_, was used to calculate the volume-weighted mean diameter, *D*[43]
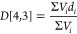
6

The size uniformity of the crystals was estimated using a span of a CSD
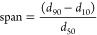
7where *d*_10_, *d*_50_, and *d*_90_ are the particle diameters at 10, 50, and 90 volumes % of cumulative distribution.

#### 2.4.2 Optical Imaging

Images of crystals of up to 400 times magnification were carried out using a Meiji microscope with a GXCAM camera (Somerset, UK).

#### 2.4.3 Scanning Electron Microscopy

High-resolution images of the crystals prepared were captured using a JSM-7800F Schottky field emission scanning electron microscope (JEOL, Japan).

#### 2.4.4 X-ray Powder Diffraction

Conformation of the crystallinity of samples as crystalline or amorphous was confirmed using a PANalytical Empyrean Series 2 diffractometer with the Bragg–Brentano geometry (University of Hull, UK).^13,24^

#### 2.4.5 FTIR Spectroscopy

The chemical identity of samples was confirmed using a Nicolet IS5 FTIR system with diamond-tipped ATR (ThermoFisher, UK).

## 3 Results and Discussion

### 3.1 Control of TEL Crystallinity

In practice, the formation of an amorphous or crystalline solid depends on how rapidly crystallization occurs at supersaturation in the antisolvent.^36,37^ In a cooling crystallization process, the rate of liquid cooling would affect whether the resulting phase is amorphous or crystalline. In the case of reverse antisolvent crystallization, the controlled mixing between the API in a solvent with the antisolvent is what determines the end outcome.

### 3.2 Stirred-Cell (LDC-1) Batch Crystallization: Crystallization without the Membrane

**Figure 4:**
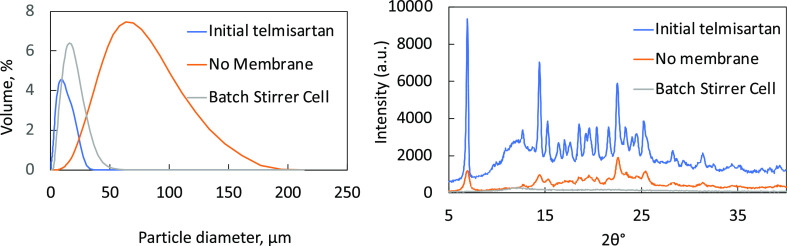
CSD curves and XRPD patterns for initially supplied TEL (crystalline), CCDC reference number XUYHOO, TEL crystallized without the membrane in LDC-1 (mixture of amorphous and crystalline) and TEL crystallized with the membrane in LDC-1 (amorphous).

To determine the importance of the membrane’s presence in the setup and its influence on the crystallization process, crystallinity, and particle size and distribution, comparative batch crystallization experiments were carried out using a stirred cell under the same conditions with and without the membrane. The resulting crystals were analyzed while in the solution to obtain CSD and crystal size, and then they were vacuum-filtered, dried, and collected for further analysis (Figure 4). CSD curves and diffraction patterns of initial TEL^47^ have been compared to those that have been crystallized via a batch stirred-cell process (LDC-1), both with and without the membrane.

The CSD curves show that without a membrane, TEL crystals and particles have been formed with a broader size distribution when compared to both the initial feedstock and TEL particles that were formed when the membrane was used. Both processes reduce the level of crystallinity compared to the original material. However, the batch stirred-cell crystallization with no membrane shows a mixture of amorphous and crystalline phases, while using the membrane confirms only the amorphous phase. The reason for the amorphous/crystalline mixture can be attributed to the removal of the membrane causing a lack of control of TEL/DMSO introduction to the DI water (antisolvent). Equations 1–3 explain how the radius of the membrane pore along with the shear stress controls the droplet size of TEL/DMSO during introduction from the membrane and how it is dispersed within the antisolvent. The mixing in the cell affects initial crystal formation, nucleation, and crystallization.

**Figure 5:**
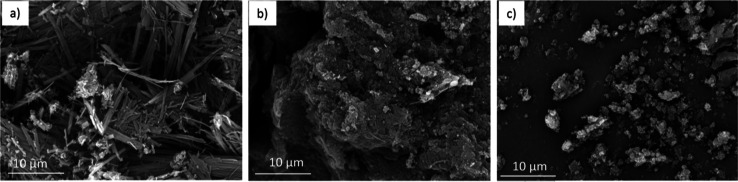
SEM images of (a) initially supplied (crystalline) TEL, (b) TEL precipitated without the membrane in LDC-1 (mixture of amorphous and crystalline), and (c) TEL precipitated with the membrane in LDC-1 (amorphous).

Using a 10 × 200 μm membrane, a TEL/DMSO concentration of 0.06 g mL^–1^, a flow rate of 10 mL min^–1^, and a stirrer speed of 1770 rpm resulted in a constant shear stress across the membrane of 24.6 Pa. The smallest TEL particles with the tightest size distribution were precipitated in the amorphous phase. By removing the membrane, the shear stress at the point of introduction of the solvent into the antisolvent was uncontrolled and inhomogeneous. This resulted in the crystallization becoming uneven and uncontrolled with nucleation and crystal growth occurring at different rates within the bulk solution. Figure 5 shows SEM images of amorphous, semicrystalline, and crystalline TEL from different crystallization methods.

The SEM images show how varied the amorphous TEL particles are when compared to crystalline needles of TEL. The second method which is that of a batch crystallization process shows a mixture of crystalline TEL needles present among amorphous TEL. This further reinforces the abilities of the micropore membrane micromixing approach to not only control crystal size and CSD but also the crystallinity.

### 3.3 Stirred-Cell (LDC-1) Batch Crystallization: Crystallization with the Membrane

According to the XRPD patterns which can be seen in Figure 3, the raw TEL prior to dissolution in the solvent (DMSO) was originally in the crystalline form. As the TEL/DMSO is introduced to the DI water antisolvent, immediate precipitation occurs. This can be attributed to the low level of TEL solubility (9.9 μg L^–1^) when in contact with DI water, which results in supersaturation, causing rapid TEL precipitation and formation of amorphous particles.^3^

Supersaturation affects both the formation of crystals (nucleation) and the crystals’ growth after formation. Once the saturation point is exceeded, nuclei will form with the rate of primary nucleation , where *K*_b_ is the nucleation constant, *C*_tel_ and *C*_tel_^*^ are the bulk concentration and solubility of TEL,  is supersaturation, and *b* is the nucleation order (for organic crystallization at 5–10).^48,49^ Once formed, crystal nuclei will grow, with the rate of crystal growth being , where *K*_g_ is the growth constant and *g* is the growth order (for organic crystallization, this value lies between 1 and 2).

**Figure 6:**
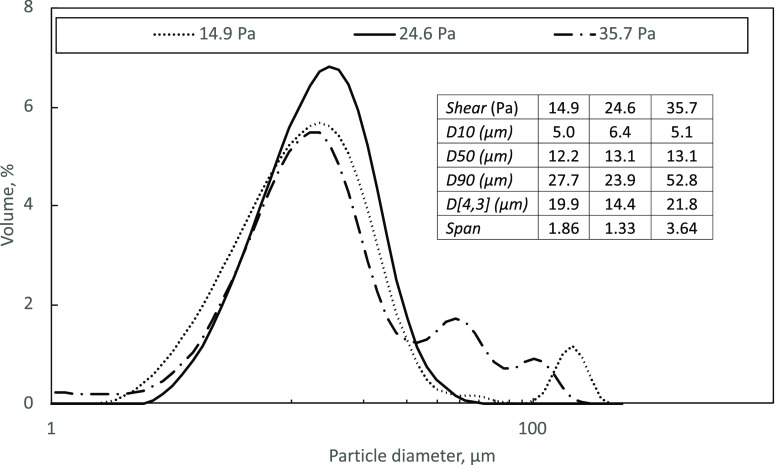
CSD curves of TEL from batch stirred-cell (LDC-1) crystallization at 14.9, 24.6, and 35.7 Pa shear from stirring. The table provides the main diameters, mean, and span of the resulting TEL particles.

A comparison of different shear stresses in Figure 6 showed that 24.6 Pa resulted in TEL particles of the most homogeneous of distributions and overall lowest sizes. Repeated batch stirred-cell runs were carried out, showing the reproducibility of the CSD curves in Figure 4c with *D*[4,3] = 15.8 ± 0.8 μm. Microscopic images of the crystals (Figure 4 before and after crystallization) showed that the amorphous material had been recrystallized from the original crystalline TEL. According to Ostwald’s rule of stages for crystal growth and nucleation, the amorphous phase has the shortest range of ordering and is the highest in free energy^43,44^ and it is also the first structure formed during the crystallization process. Stability tests showed that TEL would remain in the amorphous phase after 18 h of continuous stirring. When still in solution, it would also remain amorphous after 2 weeks in storage.

**Figure 7:**
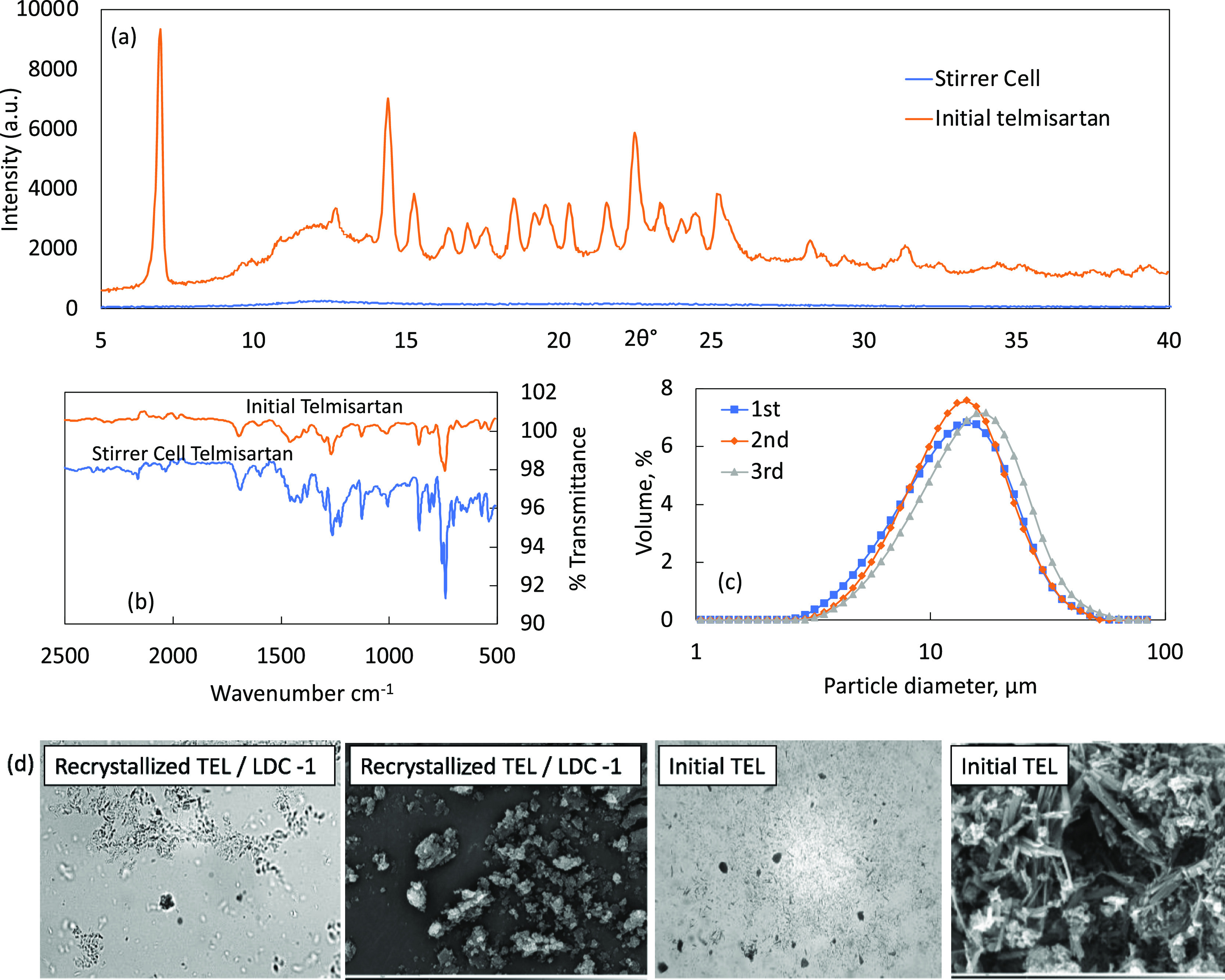
(a) XRPD patterns of initial TEL (CCDC reference number XUYHOO) and recrystallized TEL using the LDC-1 (with the membrane). (b) FTIR spectra confirming the chemical identity of initial and recrystallized TEL. (c) Reproducibility of the runs conducted in a stirred system with a 10 μm membrane, a solvent flow rate of 10 mL min^–1^, and a paddle rotation speed of 1770 rpm (corresponding shear of 24.6 Pa). (d) Corresponding optical and SEM images for initial and recrystallized TEL using LDC-1 (with the membrane).

An XRPD pattern of TEL before it was recrystallized^47^ using Micropore’s micromixing reverse antisolvent approach and after in Figure 7a confirms that the material has been transformed from crystalline to amorphous by this process. The purity of the amorphous phase was also confirmed by the FTIR spectrum in Figure 7b.^28,29^

Initial tests showed that using the standard reverse antisolvent crystallization approach with Micropore’s micromixing techniques produced amorphous TEL, confirmed by the XRPD patterns in Figure 6.^35−37^

The effect of the solubility of TEL in the antisolvent on the overall crystallization process was also investigated. Initial crystallization runs had the antisolvent as pure DI water, and as a result, amorphous TEL would immediately form. Due to the low solubility of TEL in pure DI water, the amorphous TEL is unable to dissolve to recrystallize into the more thermodynamically soluble crystalline state.

By changing the antisolvent to a mixture of DMSO and DI water (solvent and antisolvent), to a DMSO/DI ratio of 4:1, the time for crystallization to occur increased as the solubility of TEL in the antisolvent also increased. This resulted in a slower rate of crystallization and allowed the more thermodynamically stable crystalline phase to crystallize out.

### 3.4 Continuous Crossflow (AXF-1) Crystallization of TEL

After repeatable crystallization of TEL was successful using an LDC-1 (batch stirred cell) membrane contactor, the scale-up in the AXF-1 system (continuous crossflow) was then carried out. AXF-1 is designed for industrial pilot plant scale production and can be used for production rates of up to 200 L h^–1^ (outlet flow rate), and so far in the literature, it has been used for the formulation of emulsions.^40^

An advantage of using a continuous crossflow approach is that the crystals are taken away immediately and build-up of TEL on the surface of the membrane^42^ is avoided. This is particularly important for API crystallization where the formation of solids in liquids occurs; hence, build-up must be avoided to prevent membrane blockages. A continuous crossflow approach also prevents solid build-ups on surfaces that could act as seeding sites, causing inhomogeneous nucleation and resulting in a broader distribution of crystal sizes. Additionally, there is also better reproducibility as there is an immediate jump to high in-line concentration and the slow increase of the antisolvent observed in the batch stirred cell is avoided.

For scale-up crystallization, parameters used for the stirred cell were translated to the continuous crossflow system. This was aimed to prepare crystals of the same size, size distribution, and crystallinity as in the batch stirred-cell process. Additionally, this acts as a case study on how efficiently the scale-up of the crystallization process can be in general. During the continuous process, the ratio of the DP (TEL/DMSO) to CP (DI water) was 1:8 as in the batch stirred-cell runs.

**Figure 8:**
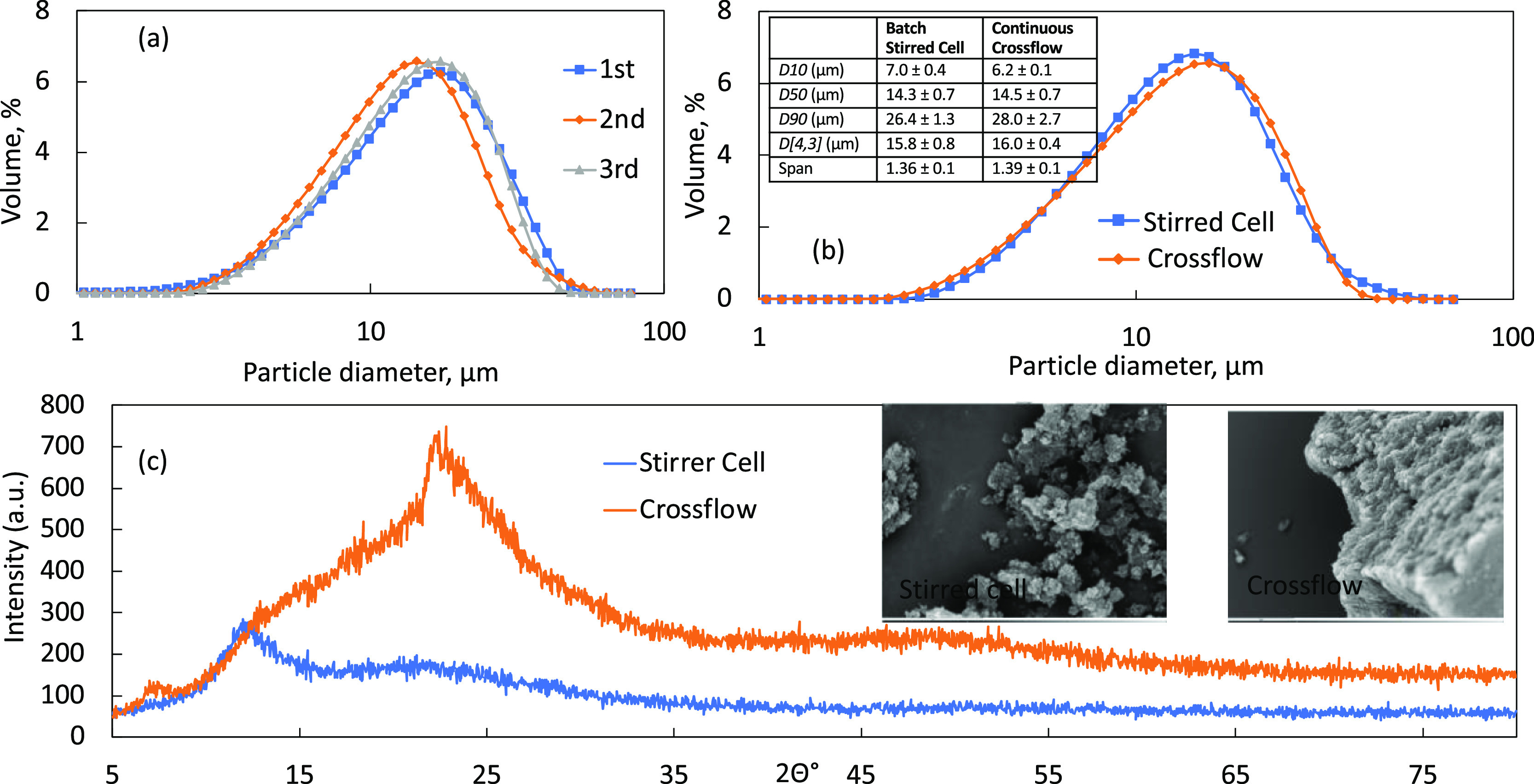
Results from crystallization of TEL using AXF-1. (a) Experimental repeatability under a shear of 24.6 Pa; CP and DP at 464 and 58 mL min^–1^, respectively. (b) Comparison of the TEL particles formed from a stirred cell and crossflow system: CSD curves and characteristic dimensions in the inserted table with average diameters/*D*-values. (c) XRPD patterns and SEM images of the amorphous material formed from LDC-1 (batch stirred cell) and AFX-1 (continuous) experiments.

In terms of what flow rates to use, the values set were those where the overall wall shear in the continuous crossflow system would be as close to that in the batch stirred cell. The wall shear in the stirred cell was at 24.6 Pa, and to replicate a value as similar to this as possible, the flow rate used was that of CP/DP of 464:58 mL min^–1^ that gave a shear wall value of 24 Pa which confirmed that the crystals were produced under the laminar flow conditions (eq 5). The average *D*[4,3] for AXF-1 runs was calculated to be 16.0 ± 0.4 μm. Figure 8a shows the CSD curves from the reproducibility runs using the crossflow.

A comparison of the CSD curves in Figure 7b and the average *D*-values (inserted table) show how scale-up reproducibility from batch to crossflow was achieved. The XRPD patterns in Figure 7c of recrystallized TEL from the different approaches also show that both crystallization methods have resulted in an amorphous product. By having the wall shear in the crossflow system close to the shear stress in the stirred cell, a similar performance of micromixing is achieved at a scaled-up level. This also indicates that the same level of supersaturation can be reached in the continuous crossflow system as in the stirred cell, with amorphous TEL being precipitated and not redissolving toward the crystalline state.^43,44^ The inset within Figure 7b provides the comparison between the diameters of TEL crystallized using an LDC-1 (stirred cell) and AXF-1 (crossflow) systems. Uncertainties for values were determined by using data from three separate runs. Using both setups, it was determined that the membrane allowed controlled rapid precipitation of the API toward the amorphous phase.

### 3.5 Effect of Membrane Pore Sizes

For the continuous crossflow system, studies on the effects of the membrane pore diameter on the crystallization process were conducted. The pores themselves are evenly dispersed in a square grid at a uniform pitch distance along the cylindrical membrane. Changing the diameter of the pores will affect the pore velocity of the DP through the membrane and as a result the laminar flow mixing. If the DP flow rate is maintained, then smaller pore sizes will result in a faster pore velocity, while larger pores will result in a slower pore velocity. Pore velocity is determined from the DP flow rate divided by the total area for the flow from the active pores. These changes in pore diameter and how they affect the mixing and precipitation process have been investigated using membrane sizes of 5, 10, 20, and 40 μm with a pitch of 200 μm. A comparison of the D-values and CSD curves from the pore sizes is shown in Figure 8 and the inset table.

By having all parameters maintained, decreasing the pore sizes from 20 to 5 μm and the resultant increase in pore velocity led to better mixing, as evidenced by the smaller average particle sizes. The crystallization of an organic system has a nucleation order greater than that of the growth order, *b* ≫ *g*, which results in growth favored at lower supersaturation with fewer larger crystals. This can be seen from the *D*[4,3] values going from 13.6 ± 0.6 to 19.9 ± 0.3 μm.

The average CSD curves show that a membrane with 5 μm resulted in crystals that had wider distribution with a distinguishable second peak, indicating that agglomeration had occurred which could be attributed to higher droplet/crystal production that resulted in agglomeration. When a 40 μm membrane was used, the crystals with a *D*[4,3] of 14.6 ± 0.8 μm were produced but with a much broader CSD compared to 10 and 20 μm membranes. The reason for this could be due to the wider pores, resulting in the streams of TEL/DMSO from individual pores spreading over the membrane, and overlapping, resulting in uneven levels of micromixing, leading to inhomogeneous supersaturation, and causing broader size distribution. As a result, the optimum pore size for achieving the tightest CSD curve and crystal sizes was found to be 10 μm.

### 3.6 Effects of DP and CP Flow Rates on the TEL Particle Size

**Figure 9:**
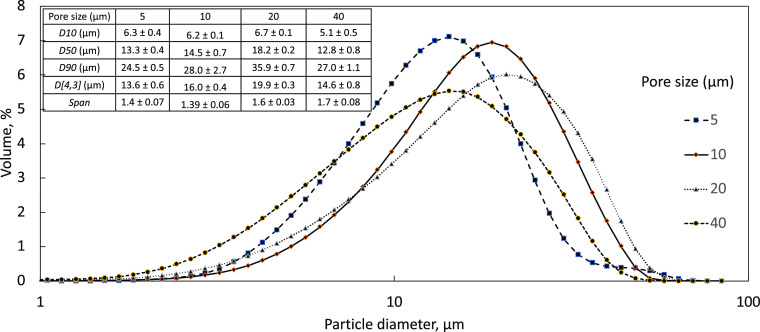
Averaged CSD curves of TEL crystallized using membranes with pore sizes of 5, 10, 20, and 40 μm with CP and DP at 464 and 58 mL min^–1^. Table with average *D*-values of TEL particles that were crystallized using membranes of different pore sizes in the continuous system. Uncertainties for values were determined by using data from three separate runs.

Changes in the DP and CP flow rates were then investigated for their effects on the nucleation and crystal growth of TEL. Initially, the CP flow rate was fixed at 464 mL min^–1^ with DP flow rates of 58, 29, and 15 mL min^–1^ investigated. The resulting *D*-values along with the CSD change along with the DP flow rates are shown in Figure 9 and the inserted table. Flow rates of 15 and 29 mL min^–1^ resulted in similar *D*[4,3] values of 11.3 ± 0.5 and 11.5 ± 0.8 μm, respectively, while a faster flow rate at 58 mL min^–1^ gave a larger particle size of 16.0 ± 0.4 μm.

Due to TEL’s poor solubility in water, when the DP first encounters the CP, supersaturation occurs on contact through micromixing. A lower DP flow rate will result in the amount of solvent in the overall flow decreasing and the volume fraction of CP increasing, leading to rapid supersaturation, which for a continuous AXF system was estimated to be 6.55 g L^–1^.^50,51^

Although a DP flow rate of 58 mL min^–1^ shows the largest *D*[4,3] value at 16.02 ± 0.6 μm, it also had the tightest CSD and the highest volumetric productivity of 6.67 mg mL^–1^ of precipitated amorphous TEL. The CSD curves for 29 and 15 mL min^–1^ showed multiple peaks, resulting in a variety of different TEL crystal sizes. This could be due to lower pore velocity, leading to inhomogeneous mixing within the stream and causing this variation in sizes.

**Figure 10:**
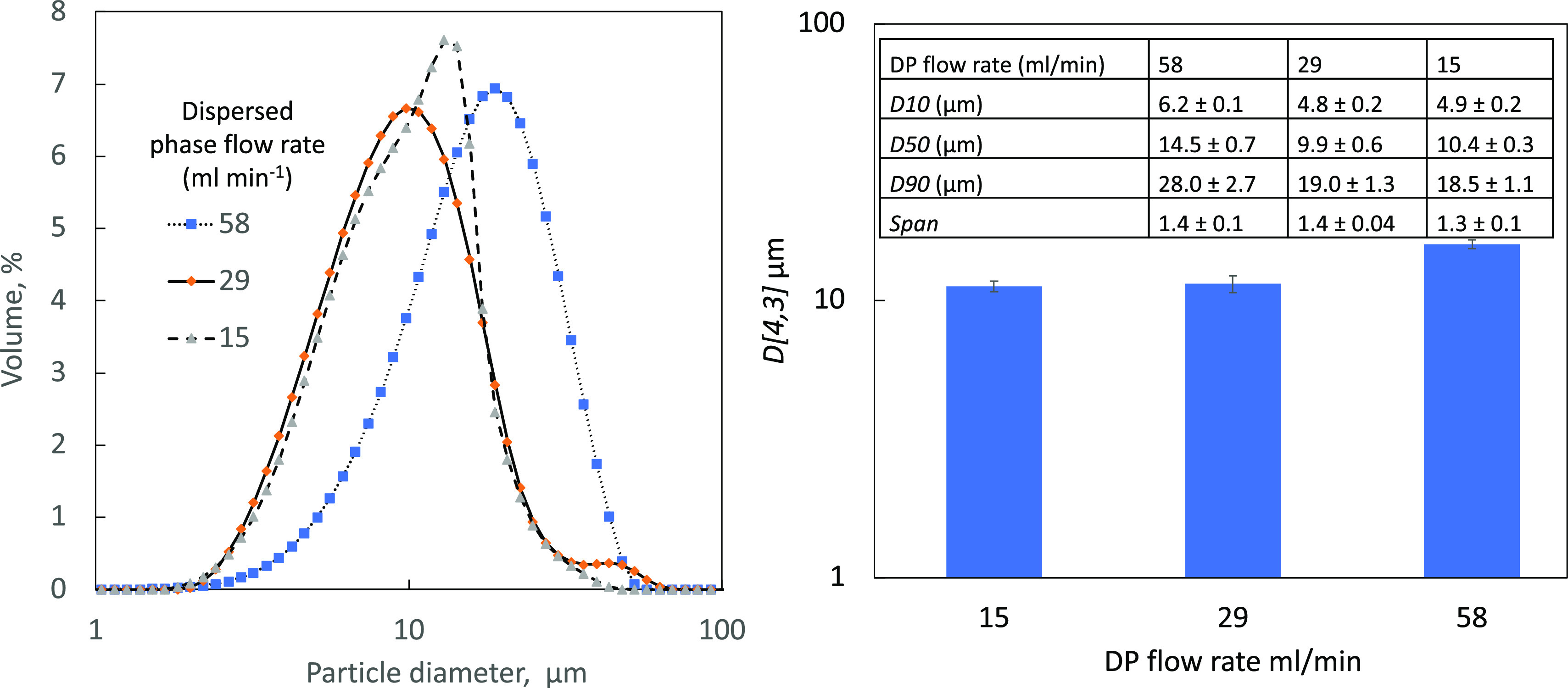
CSD curves and *D*[4,3] values of TEL crystallized with CP maintained at 464 mL min^–1^; a 10 μm membrane with a 9.5 mm insert was used. DP flow rates were varied. Table insert compares sizes of TEL particles using these various DP flow rates. Uncertainties for these values were determined by using data from three separate runs.

**Figure 11:**
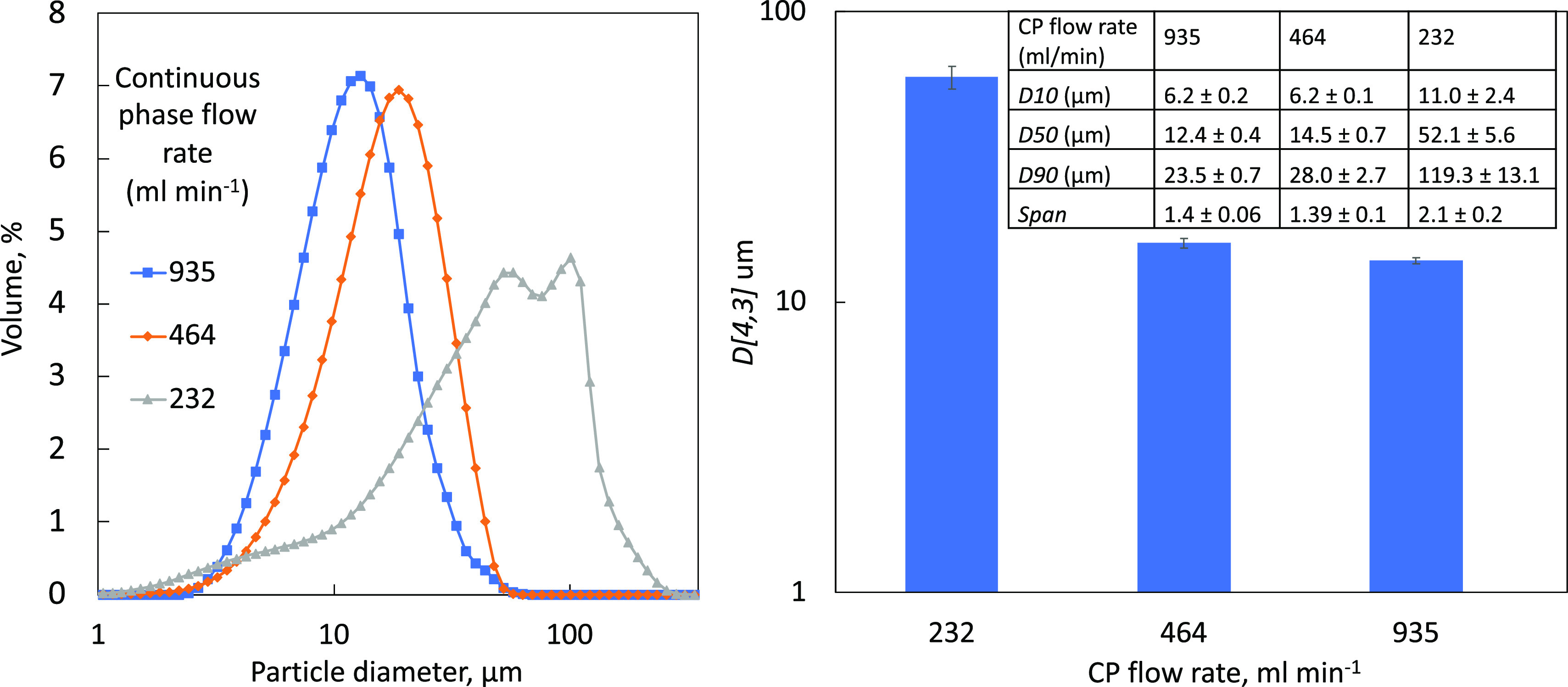
CSD curves and the effect of increasing the CP flow rate at values of 935, 464, and 232 mL min^–1^. This has a membrane with pores at 10 μm and uses a 9.5 mm insert. The table shown compares TEL particles using various CP flow rates under constant DP at 58 mL min^–1^. Uncertainties for values were determined by using data from three separate runs.

Studies in the changes in the CP flow rates at 232, 464, and 935 mL min^–1^ with the DP flow rate set at 58 mL min^–1^ and a 10 μm membrane with a 9.5 mm insert. *D*-values in the inserted table along with the comparison of CSD curves and *D*[4,3] against the CP flow rate in Figure 10 show nucleation, and thus, smaller crystal sizes are favored at higher CP flow rates.^50,51^ It is proposed that this is a result of the higher shear generated by the increased flow rates, resulting in more energetic mixing conditions and leading to faster mixing/more nucleation with less time for crystal growth and rapid removal from the membrane surface (no membrane blocking was observed). At lower CP flow rates (232 mL min^–1^), larger crystals with broad CSD curves were obtained due to slower mixing which promoted agglomeration and hence membrane blocking (Figure 11). Filipcsei et al. report that stable TEL nanoparticles, with an average particle size of less than 600 nm, can be made using a microfluidic-based continuous flow method when selected stabilizers^52^ are used. Therefore, if particles less than 1 μm are wanted, the AXF would allow the “scale-up” of the results from a microfluidic-based flow channel,^51^ but additional stabilizers^51^ would need to be added to the CP.

## 4 Conclusions

In this paper, we have presented results that show reverse antisolvent membrane crystallization in both batch stirred-cell and continuous crossflow systems toward the controlled crystallization of TEL.

Batch stirred-cell experiments showed the importance of the membrane in controlling the introduction of TEL/DMSO to the antisolvent and how this helps to determine the size and CSD of the resulting crystals, as well as the level of crystallinity. Scaling upward toward a continuous crossflow system was carried out by replicating the shear stress from the batch system.

The size of the TEL particles in the continuous-flow system was controlled by varying the solvent/antisolvent flow rate ratios and the membrane pore sizes. These allowed control of the amount of TEL/solvent that would be mixed with the antisolvent. The smallest size and CSD of amorphous TEL particles were found with pores of 10 μm and a solvent/antisolvent ratio of 0.125, and particles smaller than 1 μm could be made continuously using the continuous AXF membrane system when selected stabilizers^52^ were added to the CP.

Results from this paper prove how controlled crystallization can be achieved in a continuous crossflow system using membrane technology. This can be applied within the pharmaceutical industry to improve the solubility and reproducibility of mass-produced APIs through precise crystallization control.

## Notations

Ar: area cm^2^
*x*: droplet diameter
*r*_p_: pore radius
&tau: shear stress
&gamma: interfacial tension
*r*_trans_: transitional radius
η: dynamic viscosity of the continuous phase
ρ: continuous phase density
ω: angular velocity
δ: boundary layer thickness
*Q*: volumetric flow rate
–d*P*/d*z*: pressure gradient axially
*r*^2^: radius of the outer annulus wall
*r*^1^: radius of the inner annular wall
μ: coefficient of liquid viscosity
*R*_*e*_: Reynolds number
*D*: stirrer width
*V*_*i*_: relative volume
*i*: particles in different size classes
*d*_*i*_: mean diameter
*B*: rate of primary nucleation
*K*_b_: nucleation constant
*C*_tel_: bulk concentration of telmisartan
*C*_tel_*: solubility of telmisartan
*b*: nucleation order for organic crystallization at 5–10
*G*: rate of crystal growth
*K*_g_: growth constant
*g*: growth order for organic crystallization at 1–2
